# Reducing inappropriate psychotropic drug use in nursing home residents with dementia: protocol for participatory action research in a stepped-wedge cluster randomized trial

**DOI:** 10.1186/s12888-019-2291-4

**Published:** 2019-10-12

**Authors:** Claudia M. Groot Kormelinck, Charlotte F. van Teunenbroek, Boudewijn J. Kollen, Margreet Reitsma, Debby L. Gerritsen, Martin Smalbrugge, Sytse U. Zuidema

**Affiliations:** 1 Department of General Practice and Elderly Care Medicine, University of Groningen, University Medical Center Groningen, HPC FA21, PO Box 253, 9700 AD Groningen, The Netherlands; 2 Vilans, (Center of Expertise for Long-term Care), PO Box 8228, 3503 RE Utrecht, The Netherlands; 3 Department of Primary and Community Care, Radboud University Medical Center, Radboud Institute for Health Sciences, Radboudumc Alzheimer Center, PO Box 9101, 6500 HB Nijmegen, The Netherlands; 4 Department of General Practice and Elderly Care Medicine, Amsterdam UMC, location VUmc/Amsterdam Public Health Research Institute, PO Box 7057, 1007 MB Amsterdam, The Netherlands

**Keywords:** Inappropriate psychotropic drug use, Dementia, Nursing home, Neuropsychiatric symptoms, Implementation, Psychosocial interventions

## Abstract

**Background:**

Psychotropic drugs are often prescribed to treat neuropsychiatric symptoms in nursing home residents with dementia, despite having limited efficacy and considerable side effects. To reduce the inappropriate prescribing of these psychotropic drugs, various non-pharmacological, psychosocial, person-centered, or multidisciplinary interventions are advocated. However, existing multidisciplinary interventions have shown variable effects, with limited effectiveness often resulting from suboptimal implementation. We hypothesize that an effective intervention needs to fit the local situation of a nursing home and that support should be offered during implementation.

**Methods:**

We will embed participatory action research within a stepped-wedge cluster randomized controlled trial to study the effects of a tailored intervention and implementation plan to reduce inappropriate psychotropic drug prescribing. Nursing homes will be provided with tailored information about the perceived problems of managing neuropsychiatric symptoms and we will offer coaching support throughout. Alongside the participatory action research, we will perform a process evaluation to examine the quality of the study, the intervention, and the implementation. Our aim is to recruit 600 residents from 16 nursing homes throughout the Netherlands, with measurements taken at baseline, 8 months, and 16 months. Nursing homes will be randomly allocated to an intervention or a deferred intervention group. During each intervention stage, we will provide information about inappropriate psychotropic drug prescribing, neuropsychiatric symptoms, and difficulties in managing neuropsychiatric symptoms through collaboration with each nursing home. After this, a tailored intervention and implementation plan will be written and implemented, guided by a coach. The primary outcome will be the reduction of inappropriate prescribing, as measured by the Appropriate Psychotropic drug use In Dementia index. Secondary outcomes will be the frequency of psychotropic drug use and neuropsychiatric symptoms, plus quality of life. A mixed methods design will be used for the process evaluation. Effects will be assessed using multilevel analyses. The project leader of the nursing home and the coach will complete questionnaires and in-depth interviews.

**Discussion:**

We anticipate that the proposed tailored intervention with coaching will reduce inappropriate psychotropic drug prescribing for nursing home residents with neuropsychiatric symptoms. This study should also provide insights into the barriers to, and facilitators of, implementation.

**Trial registration:**

NTR5872, registered on July 2, 2016.

## Background

Dutch nursing homes (NHs) accommodate approximately 50,000 residents with dementia [1], and in these, the prevalence of neuropsychiatric symptoms (NPS) is high. A systematic review, for example, indicated that 82% of residents exhibited at least one NPS, with agitation and apathy being most prevalent [2]. These symptoms affect both the quality of life (QoL) of residents [3] and the health of nursing staff [4].

NPS in dementia is typically treated with psychotropic drugs, including antipsychotics, hypnotics or sedatives, anxiolytics, antidepressants, anticonvulsants, and anti-dementia drugs [5–8]. Despite the frequency with which these are prescribed, there is evidence that such drugs have limited effect on NPS in residents with dementia [9, 10] especially when used in the long-term [11]. These psychotropic drugs are also associated with significant side effects. Antipsychotics are known to increase the risk of stroke and mortality [6, 12] and to cause extrapyramidal symptoms and drowsiness [13]. The use of sedatives, hypnotics, antidepressants, and benzodiazepines is also associated with falls [14]. Together, these side effects can negatively affect QoL [10, 15–17]. Although guidelines recommend that the use of psychotropic drugs be restricted in the treatment of NPS in dementia, and although non-pharmacological alternatives are recommended for first-line treatment [18], psychotropic drugs are often prescribed in Western Europe, with antipsychotics and antidepressants being used with the greatest frequency [19]. Indeed, despite the existence of these guidelines, psychotropic drug prescribing has not substantially decreased in the Netherlands, with 60% of Dutch NH residents with dementia and NPS being prescribed at least one of these agents [20]. In addition, there is evidence that psychotropic drugs are frequently prescribed in the long-term, which again runs contrary to the guideline recommendations [21–23]. Research suggests that only 10% of psychotropic drug prescriptions for NPS are fully appropriate for residents with dementia, in terms of indication, evaluation, dosage, drug-drug interactions, drug-disease interactions, duplications, and therapy duration [20].

Several studies provide insights into the factors associated with the psychotropic drug prescribing. Relevant factors include physician and nurse attitudes to NPS and psychotropic drugs [6, 24, 25], knowledge or experience of NPS, the interpersonal skills of nurses, knowledge of the effectiveness and side effects of psychotropic drugs, communication or cooperation between professionals and with family [24], and external factors (e.g., staffing, the NH setting, and local policies) [24, 25]. However, these can only be challenged if effective non-pharmacological interventions are available, including person-centered and multidisciplinary interventions. Person-centered interventions focus on behavior, emotion, stimulation, or cognition (e.g., reminiscence, validation, music therapy, sensory stimulation) [26], whereas multidisciplinary interventions for NH staff focus on education, in-reach services, medication reviews, or multicomponent interventions to reduce inappropriate prescribing [27].

In recent decades, a number of multidisciplinary care programs have been developed to target the factors associated with inappropriate prescribing and/or to shift practice toward a greater use of non-pharmacological interventions (e.g., STA-OP, GRIP, PROPER, AiD, Dementia Care Mapping, RedUSe, and TIME) [28–34]. RedUSe is a good example of a multi-strategic interdisciplinary intervention that took place in 150 residential aged care facilities and was shown to reduce antipsychotic prescribing by 13% and benzodiazepine prescribing by 21%, without increasing their pro re nata use. Although this showed that such an intervention can be successful [34], it is generally the case that the effects of these multidisciplinary care programs or interventions are variable. In the GRIP study, for example, the effects of a multidisciplinary care program for challenging behavior were considered small, probably due to suboptimal implementation. Adjusted analyses showed larger effects in Dementia Specialized Care Units (DSCUs), in which implementation was good [29]. The results of a successful and widely implemented person-centered care approach in the United Kingdom have not been replicated in German NHs [35]. Although several changes were made to the intervention, it was thought that implementation barriers caused the loss of effect between populations. In other research, Grimshaw et al. stated that implementation studies seeking to change professional behavior achieve an effect size of 10 to 20%, with the effects likely related to the degree to which underlying barriers are addressed [36]. Striving for any culture change is challenging and takes time, and it might be unrealistic to expect larger effects. However, it is documented that a lack of effect may reflect a failure of implementation rather than a failure of the intervention itself [37, 38].

It appears that several preconditions are required for successful implementation of a new intervention, and the absence of these can effectively block implementation. Common barriers to implementation in terms of an organization’s culture have been reported to be the attitude to change and the support of key persons. Staff turnover, experience of concurrent and former projects, and organizational change have also been considered important organizational barriers [39]. By contrast, organizational preconditions for implementation are the presence of well-functioning networks, flexible organizational structures, a dementia-friendly culture, and positive attitudes of involved staff [32].

As one might imagine, creating a change in NH practice can be challenging given the complex nature of these institutions. Consequently, standardized interventions are less likely to be successful, with a need to emphasize the specific organizational features of a NH and their culture to better adapt to their specific needs [27, 32, 38, 39]. A prerequisite for successful implementation of any psychosocial intervention, whether person-centered or multidisciplinary, must therefore be that it includes some degree of tailoring. On the one hand, a person-centered intervention needs to be tailored to the preferences and abilities of a given resident [40], whereas on the other hand, a multidisciplinary program should consider the specific features of the organization and whether it fits with the needs, resources, and conditions of the NH at which it is to be implemented [32, 39]. In addition, as the complexity of an intervention increases, so too do the demands of implementation (e.g., GRIP involves multiple interacting components and requires behavioral changes in both caregivers and recipients). Complexity also varies with the possible outcomes, the number of individuals involved, and as stated, the degree to which an intervention is tailored [38]. Due to these challenges, studies of complex interventions tend to show only small to modest outcome effects [28, 29, 32, 41].

Designing interventional trials that have enough flexibility to be meaningful and successful in a local setting, without compromising generalizability, has proved challenging. In an effort to tackle this issue, Leykum et al. (2009) explored the integration of participatory action research (PAR) with a randomized controlled trial (RCT) design, successfully accounting for the local differences while creating a framework that allowed for a degree of generalizability [41]. In PAR, researchers and participants work collaboratively to define a problem, identify unmet needs, explore and implement potential solutions, and evaluate the efficacy of the implemented actions [42]. PAR aims not only to improve work practices but also to learn from the implementation process itself, helping to understand how successful implementation can be achieved. This allows the knowledge that is gained to be used to implement future complex care programs or interventions that focus on both the content and processes of the intervention. Consequently, PAR allows complex interventions to be implemented, supported by knowledge about the local NH context. According to Leykum et al. (2009), group facilitation, relationship building, and reflection should be addressed to encourage the incorporation of local conditions and contexts.

In our study, we will integrate PAR with a stepped-wedge cluster RCT to create a PAR-RCT study design. This approach will help us to account for local differences by adapting to local needs, to use a tailored approach to improve local practice, and to create a framework that allows for a degree of generalizability. A cyclic approach of planning, acting, observing, and reflecting will be used [42]. We specifically plan to address two strategies that we assume will increase the intervention’s effectiveness. First, we will provide NHs with tailored information about their perceived problems in managing NPS, including psychotropic drug use, to obtain a match between the problems experienced by NHs and the interventions to be implemented. Second, we will provide NHs with coaching to facilitate implementation. The coach will help to draft and implement the intervention plan, paying close attention to the local context of the NH. This will require dealing with any initial skepticism about non-pharmacological approaches or NH staff concerns in an effort to engender the commitment to change and the active engagement of staff that are essential for successful implementation [40].

We aim to study the effectiveness of implementing a tailored intervention to reduce inappropriate psychotropic drug prescribing in a PAR-RCT. Our goal is to change work practices, processes, and cultures at the level of DSCUs in NHs because these changes tend to have longer lasting effects [43]. Therefore, interventions chosen by the NH staff will not directly target residents, but will instead target medication reviews, behavioral visits, or the provision of information on residents’ life stories. We expect that these psychosocial interventions targeting NH staff can enhance the quality of professional conduct in NH practice, with positive effects for residents. For example, a systematic review demonstrated that studies reporting on cultural change and involving physicians may lead to a substantial reduction of antipsychotic drug prescribing [43]. It is anticipated that our intervention will lead to reductions in inappropriate psychotropic drug prescribing, the frequency of psychotropic drug use, and the frequency of NPS, as well as to an improvement in residents’ QoL. Parallel to the PAR-RCT, a comprehensive process evaluation will be conducted to provide insights into the contribution of the intervention to practice and to identify any barriers to, or facilitators of, implementation.

## Methods/design

### Design and eligibility

**Fig. 1:**
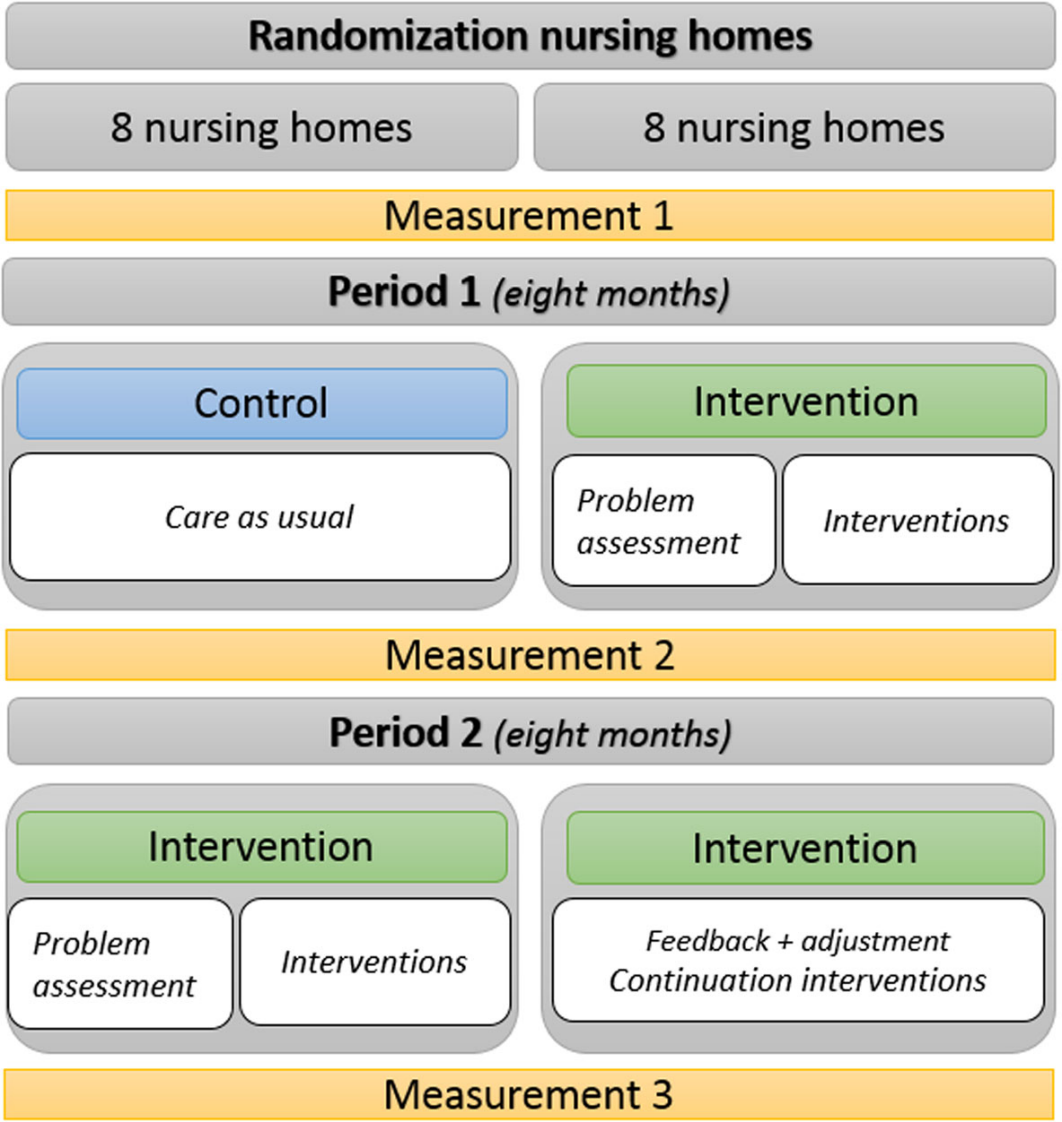
RID study design. The randomization of nursing homes into start or deferred intervention groups. *Abbreviation: RID, Reducing Inappropriate psychotropic Drug use*

We will use a two-armed cluster RCT with a stepped-wedge design to allow each NH to participate in the intervention phase and to increase the study’s power (Fig. 1). We expected that recruitment to a classic RCT design would be problematic because half do not receive the intervention, which can be off-putting. A waiting list procedure would also require including more NHs, and we want to avoid this because of the resource-intensive design in terms of time, dedication, and money. In addition to allowing for smaller sample sizes, other advantages of stepped-wedge designs are the possibility to compare both between- and within-cluster effects, as well as the ability to model effects over time [44].

The study duration will be 16 months, split into two 8-month periods. Based on a pilot study, we estimated that the problem analysis for PAR-RCT would take about 2 months. We plan to allow 6 months to implement the chosen interventions because a longer period may lead to a loss of enthusiasm among NH staff, and because we anticipate high turnover rates among residents [5] and staff [32, 39]. The coaching will also be time and cost intensive, and the amount of data collected will require a huge investment from research assistants and NH staff.

Although we believe that 8 months will be optimal for these reasons, we do recognize that it might be insufficient to bring about a change in NH practice. Therefore, we plan to use the stepped-wedge design to compare the 8 month intervention with a 16 month extended intervention that will allow us to study possible long-term effects. Using a computer program, 16 NHs will be randomized by fixed-block randomization into an intervention group or a deferred intervention group (8 NHs each). In Period 1, the intervention group (Fig. 1, green blocks) will start by implementing the PAR method, while the deferred intervention group will start with care as usual. After 8 months, Period 2 will start, and the deferred intervention group will start the intervention and the original intervention group will complete a second intervention phase.

The primary outcome will be the reduction of inappropriate psychotropic drug prescribing based on data collected at baseline, 8 months, and 16 months. The statistician will be blinded to the cluster randomization process, and the NH staff and researchers will be aware of their participation group by design. Given that we aim to intervene at the DSCU level and that residents are not directly subject to an intervention, we have no discontinuing criteria, trial stopping rules, or modifying allocations.

### Study population and recruitment

NHs will be recruited by organizing a national kick-off and using media channels to gain attention. Any interested NHs in the Netherlands can apply to participate and will be considered eligible if the board of directors and the client council agree to participate and are prepared to invest the requisite time. We will exclude NHs in which major organizational changes are expected during the study period or if other projects related to psychotropic drug use are currently running.

The study population will comprise residents with dementia who reside in psychogeriatric units (e.g., DSCUs). NHs can have several DSCUs, and several DSCUs from one NH can participate. However, DSCUs designed to deliver care for residents with Korsakov syndrome, acquired brain injury, Down syndrome, or young-onset dementia will be excluded. Although interventions will be aimed at the DSCU level, we will still need to gather data on residents to assess outcomes. Residents will be eligible to participate if they have a diagnosis of dementia according to the Diagnostic and Statistical Manual of Mental Disorders (fifth edition), have a life expectancy of at least 3 months, as judged by a physician, and provide written informed consent. We will also include users and nonusers of psychotropic drugs. It is anticipated that most residents will be at an advanced stage of dementia and may be mentally incompetent. The physician of the relevant DSCU will therefore be asked to assess a resident’s mental competence to provide informed consent. If they are not deemed competent, an employer of the registry office at each NH will send the informed consent form to a residents’ legal representative. In the absence of a response, a reminder request to return the informed consent form will be made once. If no response will be obtained, residents will not be included.

### The PAR-RCT study

**Fig. 2:**
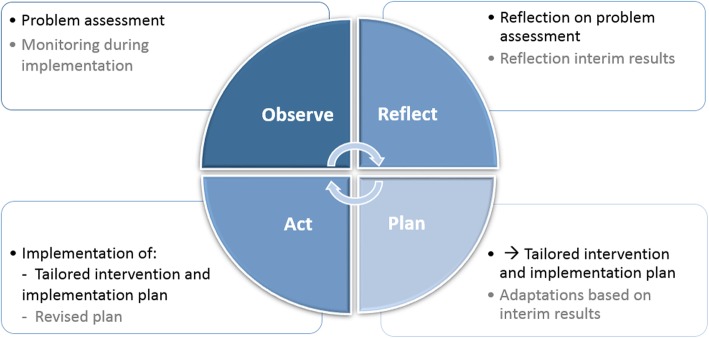
Cyclic approach of the PAR-RCT. Period 1 is shown in black and Period 2 is shown in gray. *Abbreviations, PAR, Participatory action research; RCT, Randomized Controlled Trial*

The PAR-RCT element will involve a cyclic approach of planning, acting, observing, and reflecting (Fig. 2) [42]. Half of the NHs will complete two cycles (i.e., those in the starting intervention group) and the other half of the NHs will complete one cycle (i.e., the deferred intervention group). Each NH will initially form a multidisciplinary project team (MPT) that will include, as a minimum, an internal project leader, a nursing staff representative, a psychologist, and a physician. An external coach will also participate in the MPT to facilitate the whole process.

The action research cycle will start with the researchers carrying out a problem assessment. We will start with the observation phase because effective interventions should meet the needs identified by staff [45]. This assessment will focus on current daily practice and difficulties managing NPS, including inappropriate psychotropic drug use, and the results will be presented to the MPT. Next, the MPT and the coach will reflect on these results. In this reflection phase, opportunities for improvement will be identified before the MPT moves on to formulate goals to tackle the identified problems. Two expected outcomes are predicted. First, a problem analysis may reveal that the evaluation of psychotropic drugs is inappropriate. In this instance, NHs may opt to pursue a clear work policy between physicians, to clarify the evaluation of psychotropic drugs, or to do medication reviews more frequently. Second, early detection of NPS may be identified as a problem. In this instance, they may recommend e-learning on NPS or implementing the routine use of the Neuropsychiatric Inventory-Nursing Home version (NPI-NH) by nurses for monitoring. How an NH intervenes will be decided by the coach and the MPT, with only indirect input from the researchers. The identified goals will be converted into an intervention and implementation plan. The coach will support this process and the researchers will assess whether the plan aims to reduce inappropriate psychotropic drug use. In summary, the information gained during the problem assessment will be used to formulate and operationalize the goals for the intervention and implementation plan (planning phase), after which NHs will implement the interventions (action phase).

NHs will be provided with a toolkit that contains several multidisciplinary care programs, such as GRIP or PROPER, as well as person-centered psychosocial interventions. The toolkit is a bundle of existing evidence and practices from which NHs can draw. They will be free to implement any intervention, either from the toolkit or elsewhere, provided the selected interventions match the problems identified in managing NPS and psychotropic drug use. Table 1 provides an overview of the toolkit’s content. In contrast to traditional PAR, the primary outcome (reduction of inappropriate prescribing of psychotropic drugs) is fixed in our study.

**Table 1:** Overview of the eight themes in the toolkit, including examples

*1. Leaflets, tips, and explanations*	- Collection of stories: *A pill against yelling*
*2. Training courses managing NPS*	- Alzheimer Experience ^a^ - E-learning dementia
*3. Videos about psychotropic drugs*	- Parodies on psychotropic drug use
*4. Finding alternatives*	- 85 practical alternatives to restraint - The memory suitcase ^b^
*5. Methods for NPS and depression*	- GRIP ^c^ - STA-OP ^d^ - Act in case of depression care program
*6. Improving prescription policy*	- PROPER: structured medication review - Guideline problem behavior
*7. Involving residents and relatives*	- Digital workbook: tools and materials to improve contact and cooperation between client, caregivers, and family
*8. Research, articles, publications*	*-* Dissertation GRIP study - Many articles, guidelines

^a^Film in which the viewer experiences what dementia entails from different perspectives

^b^Nostalgic suitcases full of memories and music, possibly with animation

^c^Multidisciplinary care program for managing challenging behavior

^d^Stepped care protocol for the assessment and management of pain and challenging behavior

Abbreviations: *GRIP (study)* Grip on challenging behavior, *NPS* Neuropsychiatric symptoms, *PROPER* PRescription Optimization of Psychotropic drugs in Elderly nuRsing home patients with dementia, *STA-OP (study)* serial trial intervention for pain and challenging behavior in advanced dementia patients

During the implementation phase, the MPT will have regular meetings with the coach to monitor progress (observation phase). At 8 months, NHs that start in the intervention group will receive feedback on the level of inappropriate psychotropic drug prescribing. The results of this interim analysis should allow them to reflect on changes in inappropriate prescribing (reflection phase), making it possible to adapt the intervention and implementation plan (planning phase), and act accordingly (action phase). When there is little or no reduction in the rate of inappropriate prescribing of psychotropic drugs, the MPT will be guided to consider choosing different interventions, changing their implementation strategy, or increasing the effort put into implementation.

Coaches will be responsible for the following aspects: (1) advising and supervising the internal project leader and the MPT; (2) being present at meetings of the MPT; (3) advising on the logistical aspects of the research and improvement process in the organization, including problem analysis, planning, multidisciplinary embedding, and sustainability; (4) offering substantive knowledge about psychotropic drugs, inappropriate prescribing, and reducing psychotropic drug use; (5) supporting the organization to get set up; (6) providing access to tools, methodologies, and learning networks; and (7) advising on quality assurance and dissemination of the results. We plan to recruit eight coaches through the Vilans Center of Expertise for Long-term Care, who in turn, will source them internally or through cooperating partners. The coaches must be knowledgeable about dementia and have previous consultation expertise in nursing home organizations.

Each coach will support two NHs. They may spend an average of 3 h per week with an NH over each 8-month intervention period. The 8 NHs that start in the intervention group and have an extended intervention duration will only receive coaching in the first 8 months, which will end after discussing the interim results. It is expected that time-intensive weeks will be compensated for by weeks in which little to no input is needed. Coaches will not be required to use all allocated hours, and the actual time spent with each NH is to be determined in consultation with the relevant MPT. The coaches will be asked to keep a logbook in which they will be asked to write down the number of hours spent on coaching, any agreements made with the NH, and other findings they consider relevant. They will also participate in monthly supervision sessions, led by a trainer at Utrecht University. The aim of these sessions is to discuss any difficulties experienced, to find suitable solutions, and for coaches to exchange tips. The extent to which a coach succeed will be addressed in the process evaluation.

### Process evaluation

It is essential for credibility that researchers acquire information about the quality of an intervention and its implementation in a given study [46, 47]. Leontjevas et al. (2012) proposed a process evaluation model based on first- and second-order data, which we will adopt in this study. The first-order data provide information about the internal and external validity, comprising of the sample quality (e.g., recruitment, randomization, and reach) and the intervention quality (e.g., relevance, feasibility, and extent to which an intervention was performed). The second-order data are then examined (implementation knowledge), such as implementation components that were delivered and received and the barriers to, and facilitators of, implementation. The internal project leader and coach will each receive a digital questionnaire to assess these aspects. We will follow this up with a semi-structured telephone interview to address ambiguities and to seek additional comments.

### Measurements

An overview of the outcomes and measurement instruments is provided in Table 2. Demographic data will be extracted from medical files by the researchers, including residents’ age, sex, type of dementia, pro re nata psychotropic drug use, date of last medication review, and date of admission to the DSCU. Cognitive abilities will be examined using the Cognitive Performance Scale (CPS), a reliable and valid scale that rates cognition, communication, activities of daily living and consciousness from 0 (intact) to 6 (very severe impairment) [48]. The instrument has a sensitivity and specificity of 0.94 and has been validated against the Mini Mental State Examination [49]. The CPS will be used by nursing staff, on paper, in the presence of a researcher.

**Table 2:** Overview of the measurement instruments

*Variable*	*Measurement instrument*	*Outcome measure*	*Problem analysis/ feedback MPT*
Inappropriate psychotropic drug use	APID	Primary outcome	Problem analysis + feedback 8 and 16 months ^a^
Frequency psychotropic drug use	Retrieval from medical records	Secondary outcome	Problem analysis + feedback 8 and 16 months ^a^
NPS	NPI-NH	Secondary outcome	–
	CMAI		Problem analysis
QoL	EQ-VAS ^b^	Secondary outcome	Problem analysis
	RISE		–
Current difficulties managing NPS; *NH staff*	Self-designed questionnaire [digital] + semi-structured interviews for NH staff	–	Problem analysis
Current status of managing NPS and quality of care; *relatives*	Self-designed questionnaire for relatives	–	Problem analysis
Attitude toward new interventions	EBPAS [digital] ^c^	Process evaluation	Problem analysis
Organizational culture	Questionnaire [digital] *CVF scale for long-term care* ^c^	Process evaluation	Problem analysis
Process evaluation data *model of Leontjevas* et al.*, 2012*	Self-designed questionnaire [digital] + semi-structured interview internal project leader and coach	Process evaluation	–

^a^NHs who start as an intervention group receive information on psychotropic drug use at the beginning (problem assessment), 8 months (interim results) and 16 months. NHs who start as a deferred intervention group receive information on psychotropic drug use at 8- (problem assessment) and 16 months

^b^EQ-VAS is administered to both nursing staff and relatives, at each measurement (0, 8, 16 months)

^c^The EBPAS + organization culture questionnaires are administered in the context of the problem assessment at 0 months (start intervention group) or 8 months (start deferred intervention group), as well as at 16 months in the context of the process evaluation

Abbreviations: *APID* Appropriateness of Psychotropic Prescription In Dementia, *CMAI* Cohen–Mansfield Agitation Inventory, *CPS* Cognitive Performance Scale, *CVF* Competing Values Framework, *EBPAS* Evidence-Based Practice Attitude Scale, *MPT* Multidisciplinary project team, *NPS* neuropsychiatric symptoms, *NH* Nursing homes, *NPI-NH* Neuropsychiatric Inventory-Nursing Home, *QoL* Quality of Life, *RISE* Revised Index of Social Engagement, *VAS* Visual analog scale

### Primary outcome

Psychotropic drugs will be categorized according to the Anatomical Therapeutic Chemical (ATC) classification [50]. The primary outcome is the appropriateness of psychotropic drug use, and this will be measured with the Appropriateness of Psychotropic drug use In Dementia (APID) index [23]. This index rates the prescription of regular psychotropic drugs for NPS in people with dementia, including antipsychotic, anxiolytic, hypnotic, antidepressant, anticonvulsant, and anti-dementia drugs. Psychotropic drugs administered for dementia, sleep disturbance, and delirium will be scored with the APID index, but those prescribed for psychiatric disorders will not be scored. Treatment appropriateness will be measured on seven domains: indication, evaluation, dosage, drug-drug interactions, drug-disease interactions, duplications, and therapy duration. For each domain, a score between zero (appropriate) and two (inappropriate) can be given, allowing an overall appropriateness score to be calculated (weighted sum score). The APID has been validated in residents of DSCUs in the Netherlands (intraclass correlation coefficient, 0.577–1.000) [23]. The researchers will extract information on psychotropic drug prescribing from medical records.

### Secondary outcomes

The secondary outcomes will be the frequency of psychotropic drug use, the frequency of NPS, and the QoL. The frequency of psychotropic drug use will be extracted by the researchers from medical files, and NPS and QoL will be assessed by nursing staff on paper, in the presence of a researcher. Proxy measures of NPS and QoL (nurse assessments) will be used on the assumption that residents lack capacity. NPS will be measured with the NPI-NH and the Cohen–Mansfield Agitation Inventory (CMAI). QoL will be measured using the visual analog scale (VAS) of the EQ. 5D (i.e., the EQ-VAS).

The NPI-NH measures the prevalence, frequency, severity, and associated caregiver distress of 12 neuropsychiatric symptoms. All symptoms are rated on Likert-type scales, with four-point scales used for frequency, three-point scales used for severity, and six-point scales used for caregiver distress. When a symptom is not present, the frequency, severity, and caregiver distress are not scored [51]. We plan to use the Dutch version of the NPI-NH, which has demonstrated high inter-rater agreement and validity as a rating scale [52].

The CMAI is the most used tool for determining the frequency of agitation and aggression [53]. The CMAI consists of 29 items, subdivided into three subscales: physical aggression, physically nonaggressive behavior, and verbally agitated behavior. Scores are rated on seven-point Likert-type scales, rating symptoms over the preceding 2 weeks from “never” to “several times an hour” [54]. The translated and validated Dutch version by De Jonghe and Kat (1996) will be used, which has established reliability (Cronbach’s α = 0.82) and construct validity (Inter-rater agreement = 0.89) [55–57].

The EQ-VAS records a respondent’s self-rated health on a vertical scale ranging from “best imaginable health state” to “worst imaginable health state” [58]. In our study, we will use the two proxy-versions of the EQ-VAS reported in a previous Dutch study [59]. Nursing staff and family members will be asked to rate the residents’ QoL and health status, both from their own perspective and from the perspective of the resident. In addition, the social engagement of residents will be measured as an important proxy of QoL, using the Revised Index of Social Engagement (RISE) [60]. The RISE is part of the inter-RAI Long-Term Care Facilities Assessment System and consists of six dichotomous items related to social behavior [61–63]. Its reported reliability and validity are considered sufficient [60].

### Measurements problem assessment

We will gather information on inappropriate psychotropic drug prescribing, frequency of psychotropic drug use, NPS frequency, and QoL. Also, we will analyze current difficulties in managing NPS using a self-designed, digital questionnaire for NH staff (i.e., physicians, psychologists, and nurses) to assess the following: (1) the detection, analysis, treatment, and evaluation of NPS, and (2), efforts to prevent NPS, any views about NPS, and the presence of multidisciplinary cooperation. The questionnaire will not be used to measure an outcome; instead, it will only be used to examine problems that NH staff perceive when dealing with NPS and inappropriate psychotropic drug prescribing. Scores will be rated on four-point Likert-type scales ranging from “never/rarely” to “at all times” for the frequency measure, and from “not satisfied” to “very satisfied” for the extent of satisfaction. The questionnaire has been piloted in two NHs and adjusted based on user experiences.

To gain a more in-depth insight into the processes that play a role in managing NPS and the prescription of psychotropic drugs, researchers (CGK, CvT) will conduct several semi-structured interviews with members of the MPT (e.g., physicians, psychologists, nursing staff, and managers). Several interviews were trialed in a pilot study at two NHs, and the interview formats have been adjusted according to user experience.

We also plan to address the attitudes of health care staff toward the use of new interventions or treatments. Values and beliefs on these issues influence the degree to which innovations are initiated and implemented in clinical practice [64–66], and considering the attitudes of professionals toward adopting new interventions can facilitate implementation [67]. Therefore, we will administer the Evidence-Based Practice Attitude Scale (EBPAS) developed by Aarons in 2004. This scale consists of four subscales for 15 items that are measured on five-point Likert scales, ranging from 0 (not at all) to 4 (to a very great extent). The EBPAS assesses the extent to which a professional (1) finds the intervention intuitively appealing, (2) would adopt an intervention if required by a supervisor, (3) has a general openness to trying new interventions, and (4) perceives interventions as being of limited clinical value and less important than clinical experience. We will use the Dutch version, for which the factor structure and reliability are comparable to the original version [68].

Furthermore, the organizational culture of the DSCUs will be assessed using a Competing Values Framework (CVF) scale [69]. This involves a questionnaire that consists of 24 items on four-point Likert-type scales, ranging from 1 (not characteristic) to 4 (very characteristic). It aims to provide insight into the cooperation between staff members and the working conditions and characteristics of the DSCU, and it suggests either a dominant culture type, a market type, an adhocracy type, or a hierarchy culture type. We will use a Dutch version of the questionnaire [70].

Both the EBPAS and the CVF scale will also be administered in the process evaluation, and they will be sent digitally to NH staff. Finally, a self-designed paper and pencil questionnaire will be used to assess the opinion of family members regarding the delivered care and degree of communication received with respect to NPS and/or psychotropic drug use.

### Sample size

For our primary outcome, we aim to detect a reduction in inappropriate psychotropic drug prescribing of 5 points minimum (standard deviation = 15) on the APID index from the baseline to the final measurement (16 months) [23, 71]. We expect that APID values will be nested within NHs, the main level of randomization in our design. Assuming an average size of 25 residents per NH, a power of 0.80, a significance level (alpha) of 0.05, we will need 284 psychotropic drug users. Given the multilevel design with two measurements after baseline, we anticipate that we will then need to increase this to a sample size of 364 (15 clusters) for an intraclass correlation coefficient of 0.1 [44] and a calculated design factor of 1.28 (the factor at which the original N has to multiplied). To allow for a cluster dropout of about 10% [72], and to obtain an even number of clusters, we plan to include 16 clusters with 364 residents in total. Given that approximately 60% of residents with dementia will be prescribed psychotropic drugs [20], this number will need to be further increased to 607 residents. Previous studies have shown that we can also anticipate a loss to follow-up of about 30% per year [5], which would amount to 40% in the 16 months in our study. However, in the event a resident dies or moves away from the unit, we will enroll the newly admitted resident, precluding the need to account further for attrition. Consequently, the case mix during our study will vary, and information from dropouts will need to be included in an intention to treat analysis.

### Data analysis

Primarily, we will examine results between both arms; the intervention group and the control group. Secondarily, we will examine results between the short intervention duration (8 months) and the long intervention duration (16 months). All data will be entered in a secured digital data management program. After collection, the data will be checked for outliers and extracted into a statistical software package. Descriptive statistics will be used to compare the baseline data between groups. A multilevel model will then be used to study the effects of a tailored intervention and implementation plan on reducing inappropriate psychotropic drug prescribing, based on the methods described by Twisk [73]. This model will be used for both the primary and secondary outcomes to account for the dependency of information due to the repeated measurements and cluster randomization. A restricted iterative generalized least squares algorithm will be used to estimate the regression coefficients, and the Wald test will be used to obtain a *P* value for each regression coefficient. Models will include a fixed and a random intercept.

Analyses will be adjusted for baseline outcome values (e.g., inappropriate psychotropic drug prescribing and NPS), time, and confounders (e.g., cognitive abilities, as measured with the CPS, type of dementia, distress in nurses due to NPS). We will adjust for factors that could explain why an intervention did not succeed at the team level (e.g., staff attitudes toward the use of new interventions or treatments, as measured by the EBPAS, and the cooperation between staff members, the working conditions and characteristics of the DSCU, as measured by the CVF scale). Possible interaction effects of the intervention with time and with an NH will also be investigated. Sensitivity analyses will be used to examine differences between NHs with respect to extent of performance, considering whether degree of implementation (such as more coaching) is associated with a greater reduction of inappropriate prescribing or whether attitude serves as an effect modifier. When indicated, subgroup analyses will be used to consider NHs that implement their plans unsuccessfully and NHs that implement their plans successfully. We will also conduct a missing value analysis to evaluate whether missing data are likely to be missing at random, and we will consider replacement if appropriate. Normal probability plots and plots of standardized residuals versus predicted values will be inspected to assess whether the assumptions of normality and homogeneity of variance are met. In the event of noncompliance, data transformation will be considered. The double ratings of QoL and health status, as perceived by nursing staff and family members, will be analyzed separately. Data from the process evaluation interviews will be examined in the content analysis and any barriers and facilitators will be analyzed according to the Consolidated Framework For Implementation Research [74].

## Discussion

To the best of our knowledge, no study has used a PAR-RCT design to examine the effect of a tailored intervention and implementation plan on the reduction of inappropriate psychotropic drug prescribing for NH residents with dementia. We consider the study design to be a strength of this proposal because active participation of NH staff (those most involved in the care process) is most likely to engender engagement and intervention suitability in the long-term [41]. In addition, the use of coaching should ensure that implementation conditions are optimal by ensuring that close attention is paid to the local NH context, including staff commitment [40].

We anticipate that the results of our study will provide evidence for the effectiveness of a tailored intervention and implementation plan. Additionally, the results should offer insights into the issues surrounding the implementation of complex interventions in NHs, including relevant barriers and facilitators, which can be accounted for in future implementation processes. The use of a stepped-wedge design offers several advantages, such as the possibility of between- and within-group analyses, increased study power, and of comparing long- and short-term intervention durations [44]. Given that the design also ensures that each NH receives the intervention, unlike in a standard RCT with a regular control group that does not receive the intervention, we also anticipate that this will enhance motivation in the participating NHs [75].

A few limitations also warrant mention, such as NHs not being randomly selected and participation being on a voluntary basis, which could introduce selection bias. NHs and researchers will also be aware of the condition (deferred intervention or intervention), which might increase the likelihood of bias. In our sample size calculation, we use an effect size of 5 points on a scale ranging from 0 to 102.8. Although this was required to show a significant effect in favor of the intervention group in an earlier study, there is insufficient literature to determine whether this is clinically relevant. The APID index measures the appropriateness of psychotropic drug use based on medical records, and its “indication” and “evaluation” items have low inter-rater agreement, largely due to bias in medical record extraction. In addition, scoring the APID index can be affected by the quality of reporting. Suboptimal recordkeeping, such as not reporting an indication, could influence the score. Keeping medical files up to date is therefore essential for accurate judging of the appropriateness of prescribing [23].

Other limitations include the difficulty inherent to examining the effect of an implemented intervention. In the tailored intervention and implementation plan with coaching, we can only evaluate the PAR-RCT design as a whole to help local practice with current knowledge tailored to specific needs. We will be unable to state whether an intervention in NH “A” (e.g., GRIP) was more or less effective than that in NH “B” (e.g., medication review), because each NH will be at liberty to choose its own interventions. At best, the sensitivity analyses will be able to show whether the degree of implementation (e.g., more coaching) was associated with a greater reduction in inappropriate psychotropic drug prescribing or whether attitudes better serve as an effect modifier. Also, secondary outcomes (e.g., NPS and QoL) will be measured based on proxy reports by nursing staff, which may be less reliable than direct measures. To account for this, we will use common observation scales for NPS (e.g., the NPI-NH and CMAI). Reporting bias may also occur for the QoL measures [75]. Therefore, to enhance the credibility of the results in these domains, only NH staff who are frequently involved in the daily care of a given resident will be asked to complete the questionnaires.

## Data Availability

Not applicable.

## Abbreviations

*AiD*: Act in case of Depression
*APID*: Appropriate Psychotropic drug use In Dementia
*CMAI*: Cohen–Mansfield Agitation Inventory
*CPS*: Cognitive Performance Scale
*CVF*: Competing values framework
*DSCU*: Dementia Specialized Care Unit
*EBPAS*: Evidence-Based Practice Attitude Scale
*GRIP*: Grip on challenging behavior
*MPT*: Multidisciplinary project team
*NH*: Nursing homes
*NPI-NH*: Neuropsychiatric Inventory-Nursing Home
*NPS*: Neuropsychiatric symptoms
*PAR*: Participatory action research
*PAR-RCT*: PAR with a stepped-wedge cluster RCT
*PROPER*: PRescription Optimization of Psychotropic drugs in Elderly nuRsing home patients with dementia
*QoL*: Quality of life
*RCT*: Randomized Controlled Trial
*RedUSe*: Reducing Use of Sedatives
*RID*: Reducing Inappropriate psychotropic Drug use
*RISE*: Revised Index of Social Engagement
*STA-OP*: Serial trial intervention for pain and challenging behavior in advanced dementia patients
*TIME*: Targeted Interdisciplinary Model for Evaluation and Treatment of Neuropsychiatric Symptoms
*UMC*: University Medical Center
